# Prognostic impact of an integrative analysis of [^18^F]FDG PET parameters and infiltrating immune cell scores in lung adenocarcinoma

**DOI:** 10.1186/s13550-022-00908-9

**Published:** 2022-06-27

**Authors:** Jinyeong Choi, Azmal Sarker, Hongyoon Choi, Dong Soo Lee, Hyung-Jun Im

**Affiliations:** 1 Department of Applied Bioengineering, Graduate School of Convergence Science and Technology, Seoul National University, Seoul, 08826 Republic of Korea; 2 Department of Nuclear Medicine, Seoul National University Hospital, Seoul, Republic of Korea; 3 Department of Molecular Medicine and Biopharmaceutical Sciences, Graduate School of Convergence Science and Technology, Seoul National University, Seoul, 08826 Republic of Korea; 4 Cancer Research Institute, Seoul National University, 03080 Seoul, Republic of Korea; 5 Research Institute for Convergence Science, Seoul National University, Seoul, 08826 Republic of Korea

**Keywords:** Radiogenomics, Lung adenocarcinoma, ^18^F-FDG PET, Transcriptomics

## Abstract

**Background:**

High levels of ^18^F-fluorodeoxyglucose (^18^F-FDG) tumor uptake are associated with worse prognosis in patients with non-small cell lung cancer (NSCLC). Meanwhile, high levels of immune cell infiltration in primary tumor have been linked to better prognosis in NSCLC. We conducted this study for precisely stratified prognosis of the lung adenocarcinoma patients using the integration of ^18^F-FDG positron emission tomography (PET) parameters and infiltrating immune cell scores as assessed by a genomic analysis.

**Results:**

Using an RNA sequencing dataset, the patients were divided into three subtype groups. Additionally, 24 different immune cell scores and cytolytic scores (CYT) were obtained. In ^18^F-FDG PET scans, PET parameters of the primary tumors were obtained. An ANOVA test, a Chi-square test and a correlation analysis were also conducted. A Kaplan–Meier survival analysis with the log-rank test and multivariable Cox regression test was performed to evaluate prognostic values of the parameters. The terminal respiratory unit (TRU) group demonstrated lower ^18^F-FDG PET parameters, more females, and lower stages than the other groups. Meanwhile, the proximal inflammatory (PI) group showed a significantly higher CYT score compared to the other groups (*P* = .001). Also, CYT showed a positive correlation with tumor-to-liver maximum standardized uptake value ratio (TLR) in the PI group (*P* = .027). A high TLR (*P* = .01) score of ^18^F-FDG PET parameters and a high T follicular helper cell (TFH) score (*P* = .005) of immune cell scores were associated with prognosis with opposite tendencies. Furthermore, TLR and TFH were predictive of overall survival even after adjusting for clinicopathologic features and others (*P* = .024 and .047).

**Conclusions:**

A high TLR score was found to be associated with worse prognosis, while high CD8 T cell and TFH scores predicted better prognosis in lung adenocarcinoma. Furthermore, TLR and TFH can be used to predict prognosis independently in patients with lung adenocarcinoma.

**Supplementary Information:**

The online version contains supplementary material available at 10.1186/s13550-022-00908-9.

## Background

Lung cancer is the most common cause of cancer death worldwide [1]. Non-small cell lung cancer accounts for 85% of all cases, and adenocarcinoma is the most common pathological subtype [2]. Currently, TNM staging according to the American Joint Committee on Cancer (AJCC) guideline is used for the stratification of lung adenocarcinoma to predict clinical outcomes and to decide upon treatment strategies; however, the treatment responses and prognoses of patients rated at the same TNM stage vary widely [3–5]. Therefore, research leading to the development of more accurate methods beyond TNM staging for patient stratification in lung adenocarcinoma is actively underway [6–8].

^18^F-fluorodeoxyglucose positron emission tomography/computed tomography (^18^F-FDG PET/CT) is a useful tool for the differential diagnosis, staging and prediction of clinical outcomes in non-small cell lung cancer (NSCLC) [9]. Specifically, quantitative parameters such as the maximum standardized uptake value (SUVmax), metabolic tumor volume (MTV) and total lesion glycolysis (TLG) as obtained from ^18^F-FDG PET/CT were shown to be prognostic in previous meta-analyses [10, 11]. However, ^18^F-FDG uptake associated with the tumor is determined by both cancerous cells and infiltrating immune cells [12, 13]. Accordingly, it would be beneficial to consider the influence of infiltrating immune cells when utilizing ^18^F-FDG uptake levels to predict clinical outcomes.

Tumor-infiltrating immune cells play a critical role in tumor development and progression. The dynamic interaction between tumor cells and tumor-infiltrating immune cells results in both host-protective and tumor-promoting effects [14, 15]. Consequently, infiltrating immune cells have an impact on the prognosis of various types of malignancies, including lung cancer [16–19]. Specifically, in recent meta-analyses, CD3 + , CD4 + , CD8 + and CD20 + T cells were associated with favorable prognosis in NSCLC, whereas FOXP3 + regulatory T cells were associated with poor prognosis [20, 21]. In addition, tumor-infiltrating immune cells have been investigated as predictive biomarkers for immune checkpoint inhibitors [22].

Herein, we hypothesized that clinical outcomes of patients with lung adenocarcinoma can be precisely predicted by an integrative analysis of ^18^F-FDG PET parameters and infiltrating immune cell scores. To that end, we assessed the prognostic value of combining immune cell scores quantified by a RNA sequencing analysis and metabolic parameters calculated by ^18^F-FDG PET studies. Also, we more closely examined the association between 1) ^18^F-FDG PET parameters and molecular subtypes of adenocarcinoma and 2) ^18^F-FDG PET parameters and immune cell scores.

## Methods

### Data acquisition

**Fig. 1:**
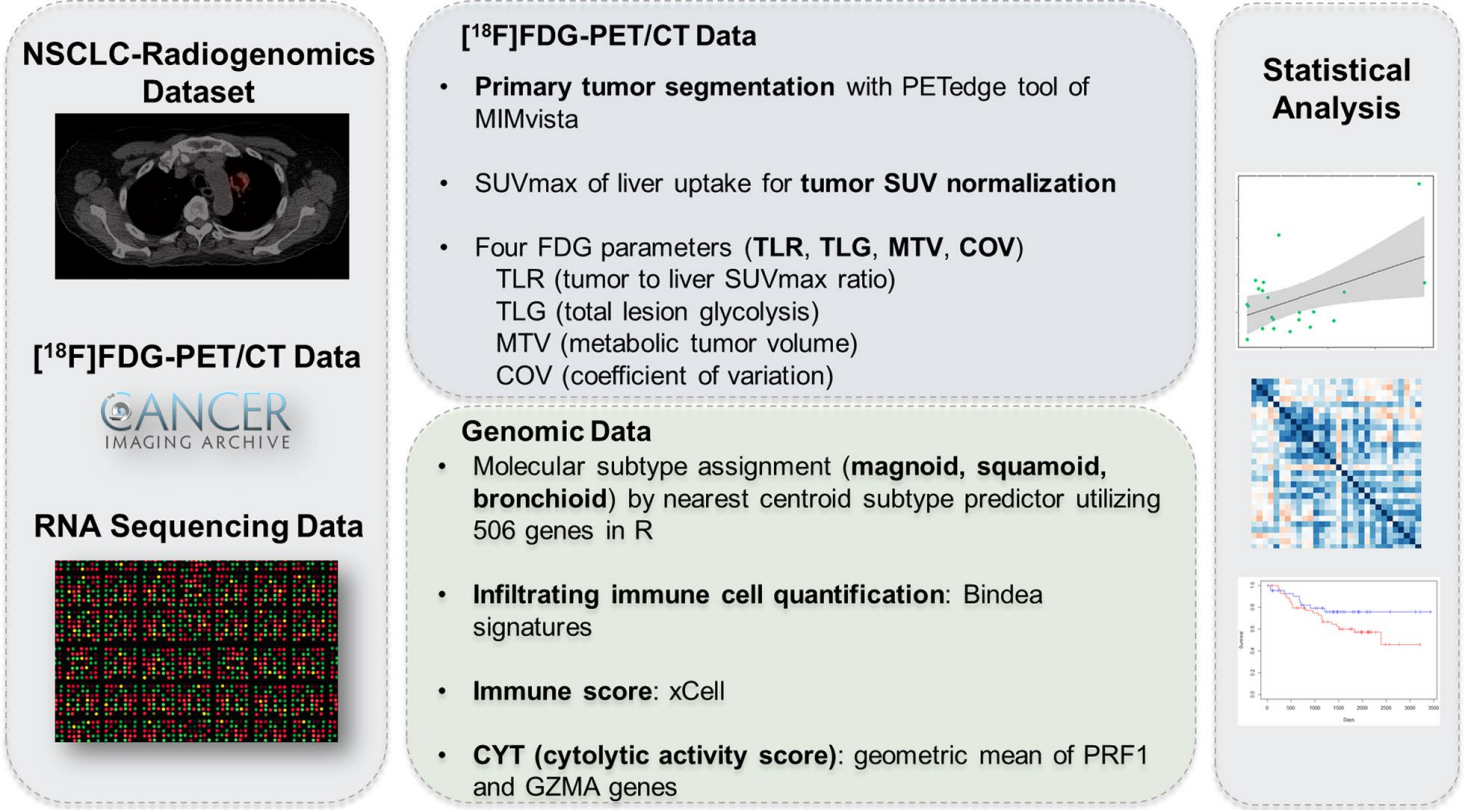
Scheme of the study. ^18^F-FDG PET and RNA sequencing data were obtained from NSCLC radiogenomics dataset in TCIA. Four ^18^F-FDG parameters were measured from manually assigned ROI of primary tumor. RNA sequencing data used for molecular subtype assignment and immune-related quantification. Statistical analysis was performed by integrating ^18^F-FDG parameters and genomic signatures

The overall content of the research is presented in Fig. 1. All RNA sequencing, ^18^F-FDG PET and clinical data were obtained from the NSCLC radiogenomics dataset in The Cancer Imaging Archive (TCIA) [23]. Medical images and TCIA data are available for public download without patient identifiers. These data were collected with patients’ agreement as approved by the institutional review boards of all participating institutions following the 1964 Helsinki declaration and its later amendments or comparable ethical standards. Among the non-small cell lung cancer data for 130 patients, 96 lung adenocarcinoma patients in total were available with regard to ^18^F-FDG-PET/CT data and RNA sequencing data.

### Tumor metabolic parameters of ^18^F-FDG PET

For primary tumor segmentation, the PETedge tool of MIMvista (MIM Software Inc., USA) was used by a specialist. The ^18^F-FDG parameters of the maximum standardized uptake value (SUVmax), metabolic tumor volume (MTV) and total legion glycolysis (TLG) were obtained from primary tumor segmentation. Also, the SUVmax score of the liver was calculated from each patient for normalization by drawing a 3.0-mm-diameter globular region of interest (ROI). The tumor-to-liver SUVmax ratio (TLR) was defined as the tumor SUVmax value divided by the liver SUVmax value [24, 25]. The coefficient of variation (COV) is measured as the ratio of the standard deviation to the mean, which can be considered as a feature to reflect metabolic heterogeneity [19].

### Molecular subtype assignment and immune cell scores by transcriptomic data

In the molecular subtype assignment step, we classified 96 patients into categories referred to here as proximal proliferative (PP, formerly magnoid), terminal respiratory unit (TRU, formerly bronchioid) and proximal inflammatory (PI, formerly squamoid) using the nearest centroid subtype predictor with 506 genes in R (version 3.4.4), referring to work by Wilkerson et al. [26]. First, we filtered 506 gene expression values consistent with subtype predictor genes from lung adenocarcinoma patients, after which the values were median-centered. Second, correlation coefficients were calculated by the Pearson correlation method between individual patients and the centroids of each subtype. Finally, the subtype with the maximum correlation coefficient was assigned for patients.

Immune cell scores for 28 different types of immune cells were calculated using RNA sequencing data according to the method by Bindea et al. [27]. Cytolytic activity scores (CYT) were obtained as the geometric mean of the perforin-1 gene (PRF1) and granzyme A gene (GZMA) [28].

### Statistical analysis

Comparisons of clinical variables, the cytolytic score and the ^18^F-FDG PET parameters according to the molecular subtype were done by Chi-square tests and ANOVA tests. A correlation analysis between the ^18^F-FDG PET parameters, immune signatures and immune cells was conducted by means of a Pearson correlation analysis. The patients were divided into low and high groups according to the median value of each continuous variable for a survival analysis. In the survival analysis, high and low groups were assessed by a Kaplan–Meier survival analysis and a log-rank test. To assess the stratified prognostic value further, a Cox multivariate regression analysis was done using the ^18^F-FDG PET parameters and immune cells. All statistical analyses were two-sided, and P values less than 0.05 were regarded as significant. These analyses were performed with R (version 3.4.4) and SPSS (version 25).

## Results

### Patients’ characteristics

The characteristics of the ninety-six lung adenocarcinoma patients with both ^18^F-FDG PET and RNA sequencing data are described in Table 1. The median age of the patients was 68 years, and the median follow-up period was 1399 days. The proportion of male patients (*n* = 67) was about 2.3 times higher than that of female patients (*n* = 29), and seventy-five patients (approximately 78%) had a history of smoking. Clinical staging was done according to the 8th edition of the AJCC, and 76% of the patients were classified as clinical stages I and II. The most prevalent location of the primary tumor was right upper lobe, affecting 33 patients.

**Table 1:** Patient characteristics

Patients, *n*	96
Median follow-up (days)	1399 (19–3433)
*Vital status*	
Dead	31 (32.3%)
Alive	65 (67.7%)
*Age (years)*	
Median	68
Range	43–85
*Gender*	
Male Female	67 (69.8%) 29 (30.2%)
*Clinical stage*	
0 I II III IV	5 (5.2%) 55 (57.3%) 18 (18.8%) 13 (13.5%) 5 (5.2%)
*Tumor site*	
RUL RML RLL LUL LLL L Lingula	33 (34.4%) 9 (9.4%) 12 (12.5%) 25 (26.0%) 16 (16.7%) 1 (1.0%)
*Smoking status*	
Nonsmoker Former Current	21 (21.9%) 53 (55.2%) 22 (22.9%)
*Mutation status (EGFR)*	
Wild type Mutated Unknown	63 (65.6%) 22 (22.9%) 11 (11.5%)
*Mutation status (KRAS)*	
Wild type Mutated Unknown	61 (63.5%) 19 (19.8%) 16 (16.7%)

### Association between molecular subtypes and clinical variables, immune scores and ^18^F-FDG PET parameters

**Fig. 2:**
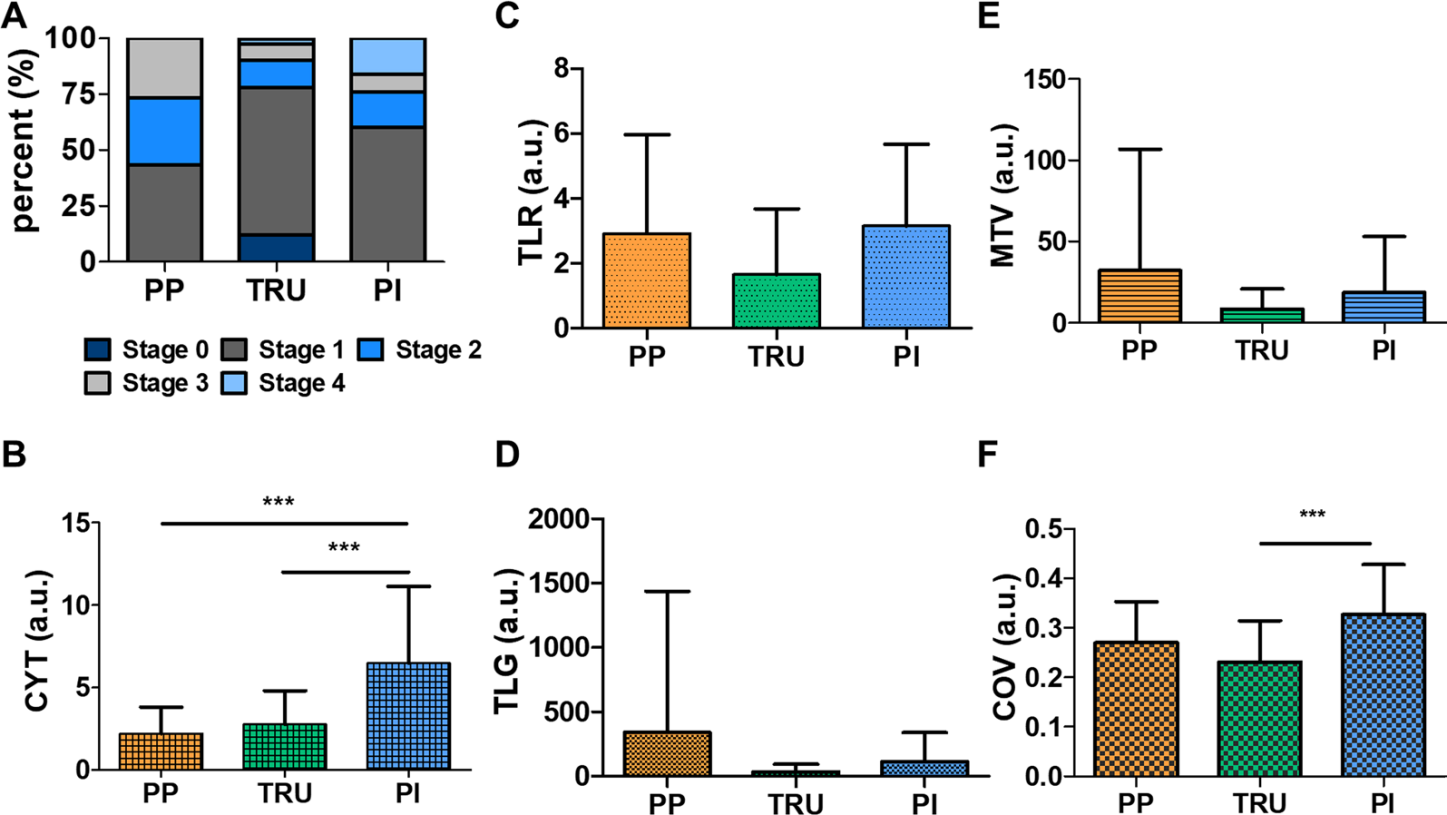
Correlation between molecular subtypes, stages, CYT and ^18^F-FDG parameters: **A** the percent of each stage is represented in bar graphs according to each molecular subtype. **B–F** The scores of the CYT and ^18^F-FDG parameters are represented in bar graphs with the error bar by each molecular subtype. Three asterisks (***) mean *P* value < 0.001

The patients were grouped according to the molecular subtype (PP, TRU and PI) based on RNA sequencing data, with 30, 41 and 25 patients in each group, respectively. We examined whether there were differences in clinical variables and immune scores between the three groups. We found that sex and stage distribution were significantly different among the three groups (*P* = 0.004, and 0.002, respectively). In a post hoc analysis, the proportion of women was significantly higher in the TRU group than in the PI group (*P* = 0.002). Also, the TRU group contained more early stage patients compared to the PP and PI groups (*P* = 0.02, and 0.043, respectively) (Table 2, Figure A). Meanwhile, the CYT score of the PI group was significantly higher than those of the PP and TRU groups (*P* = 0.001, and 0.001, respectively), which indicates that the degree of anticancer immunity is highest in the PI group (Table 2, Fig. 2B). We also compared the ^18^F-FDG PET parameters between the groups, finding that TLR and COV were significantly different between the groups (*P* = 0.038 and 0.001, respectively). In a post hoc test, TLR tended to be higher in the PI group than in the TRU group (*P* = 0.061). Moreover, the PI group had significantly higher COV scores than those of the TRU group (*P* = 0.001) (Table 3, Fig. 2C-F). Taken together, the PI group demonstrated the highest levels of anticancer immunity, ^18^F-FDG uptake and metabolic heterogeneity among the three groups. Given that ^18^F-FDG uptake can be determined by both infiltrating immune cells and malignant cells, we explored the association between the ^18^F-FDG PET parameters and immune cell scores further.

**Table 2:** Correlation between molecular subtypes with clinical variables and immune signatures

	Group			ANOVA or Chi-square	Post hoc test	
	PP (*n* = 30)	TRU (*n* = 41)	PI (*n* = 25)	*P* value	Comparison	*P* value
Age	67.23 ± 8.581	66.63 ± 11.083	69.52 ± 8.804	0.499	PP vs. TRU	0.965
					TRU vs. PI	0.479
					PI vs. PP	0.665
Sex (% of F/M)	26.7/73.3	46.3/53.7	8/92	0.004	PP vs. TRU	0.154
					TRU vs. PI	0.002
					PI vs. PP	0.263
Stage (% of 0/I/II/III/IV)	0/43.3/30/26.7/0	12/65.9/12.2/7.3/2.4	0/60/16/8/16	0.002	PP vs. TRU	0.020
					TRU vs. PI	0.043
					PI vs. PP	0.990
CYT	2.21 ± 1.59	2.75 ± 2.06	6.47 ± 4.68	0.001	PP vs. TRU	0.822
					TRU vs. PI	0.001
					PI vs. PP	0.001

*TRU* terminal respiratory unit, *PP* proximal proliferative, *PI* proximal inflammatory, *CYT* cytolytic score

**Table 3:** Correlation between molecular subtypes and ^18^F-FDG parameters

	Group			ANOVA or Chi-square	Post hoc test	
	PP (*n* = 30)	TRU (*n* = 41)	PI (*n* = 25)	*P* value	Comparison	*P* value
TLR	2.91 ± 3.05	1.65 ± 2.03	3.15 ± 2.52	0.038	PP vs. TRU	0.104
					TRU vs. PI	0.061
					PI vs. PP	0.935
MTV	32.19 ± 74.59	8.41 ± 12.44	18.66 ± 34.36	0.133	PP vs. TRU	0.111
					TRU vs. PI	0.693
					PI vs. PP	0.558
TLG	340.88 ± 1096.37	34.07 ± 57.90	111.89 ± 227.86	0.154	PP vs. TRU	0.140
					TRU vs. PI	0.892
					PI vs. PP	0.411
COV	0.270 ± 0.083	0.231 ± 0.084	0.327 ± 0.101	0.001	PP vs. TRU	0.177
					TRU vs. PI	0.001
					PI vs. PP	0.057

*TRU* terminal respiratory unit, *PP* proximal proliferative, *PI* proximal inflammatory, *CYT* cytolytic score, *TLR* tumor-to-liver SUVmax ratio, *MTV* metabolic tumor volume, *TLG* total lesion glycolysis, *COV* coefficient of variation

### Correlation between ^18^F-FDG PET parameters and immune cell scores

**Fig. 3:**
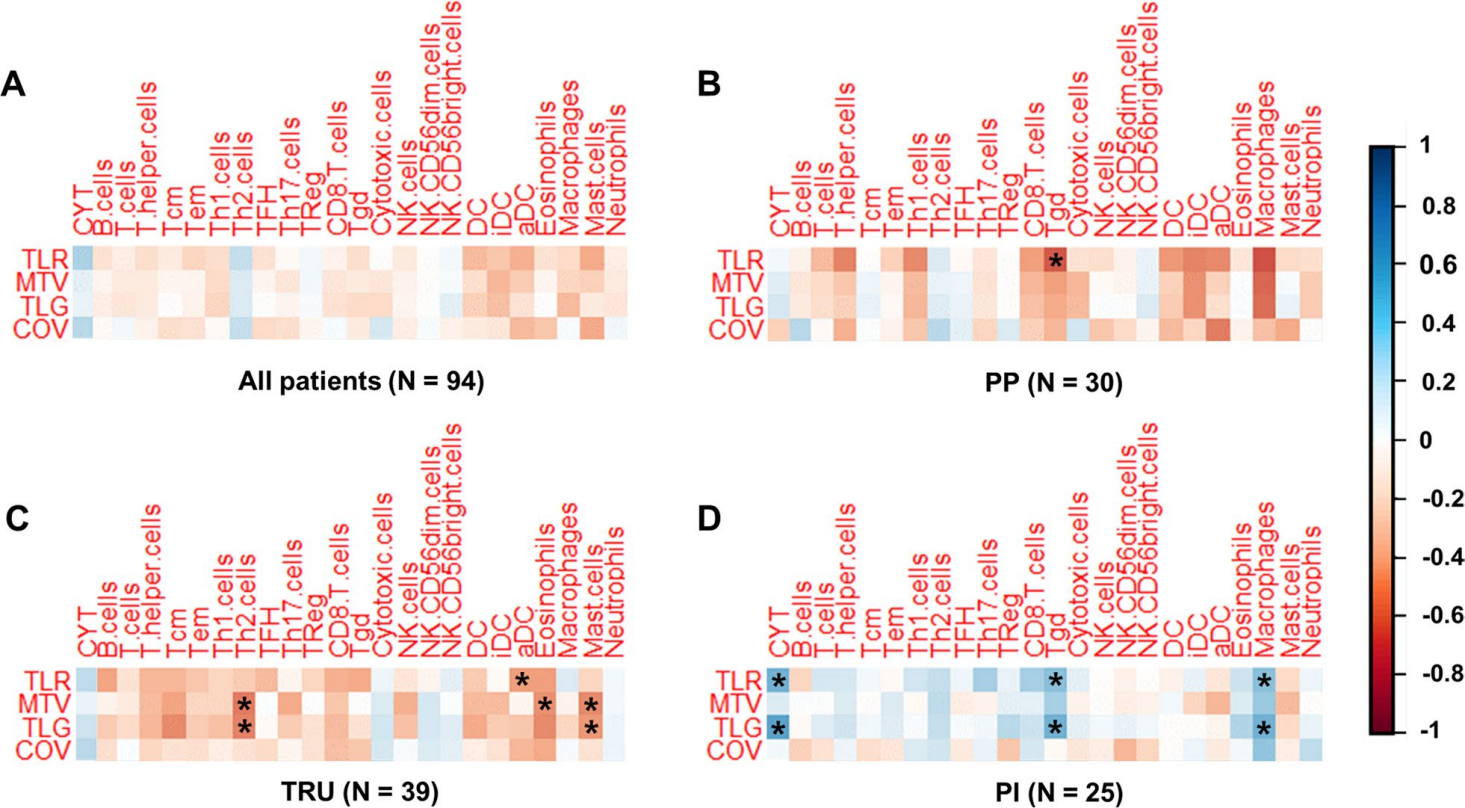
Correlation map of ^18^F-FDG parameters and immune cell scores in all patients and each molecular subtype. Correlation coefficients (*r*) are expressed using a color scale. An asterisk means a pair for which |*r*|> 0.4 and *P* < 0.05. This result has not corrected for multiple testing. **A** correlation map of ^18^F-FDG parameters and immune cell scores in all patients, **B**–**D** correlation map of ^18^F-FDG parameters and immune cell scores in PP, TRU and PI group

**Fig. 4:**
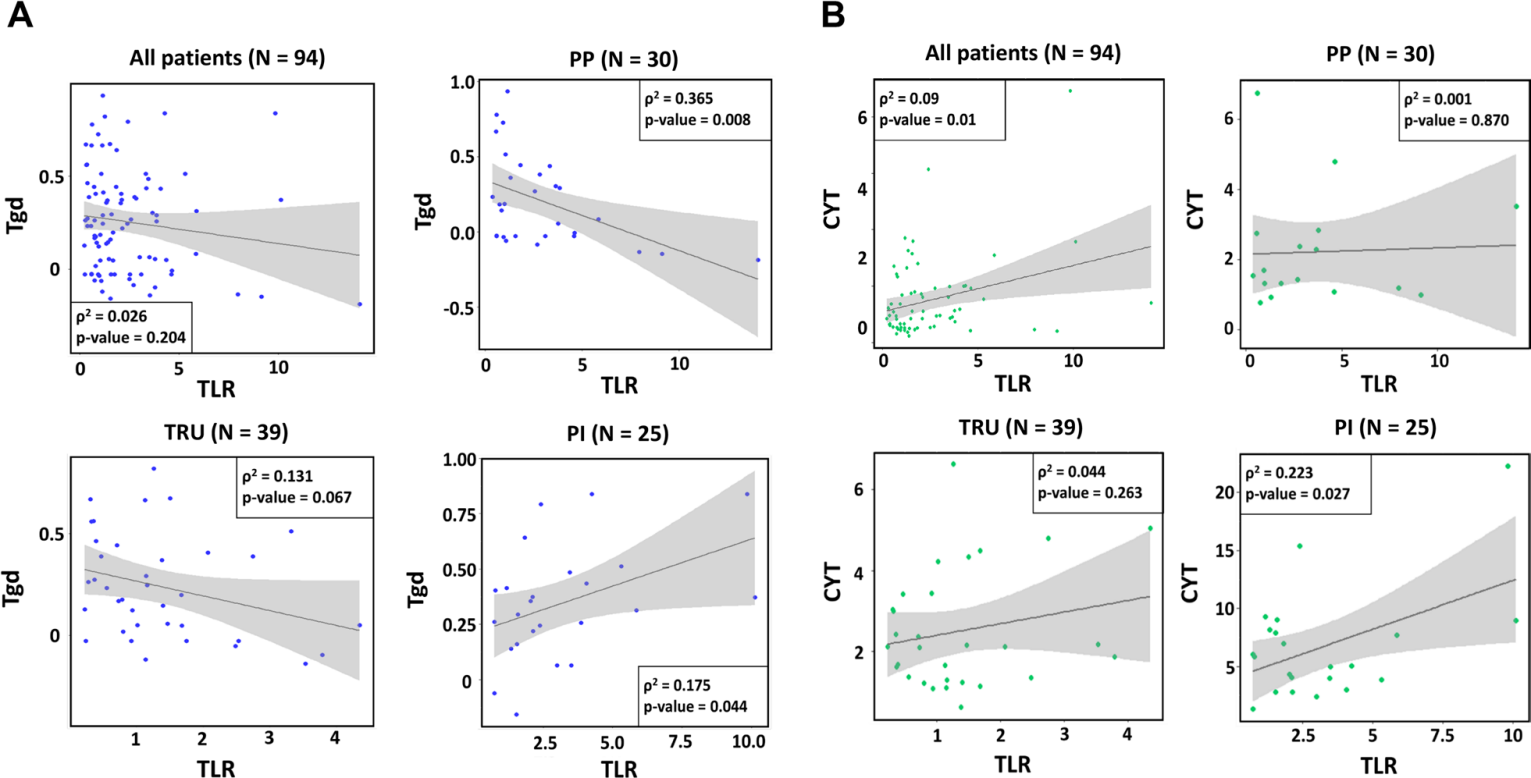
Correlation between ^18^F-FDG parameter and immune cells: **A** scatter plots for a correlation analysis of TLR and Tgd, and **B** scatter plots for a correlation analysis of TLR and CYT. The boxes in each scatter plot show the coefficient of determination and the p value. The dark gray line is a linear regression line, and the gray region is the 95% confidence region

A correlation analysis was conducted considering the ^18^F-FDG PET parameters, CYT scores and immune cell scores. With a pair for which absolute value of correlation coefficient |r|> 0.4 and *P* < 0.05 considered to be significant, there were no pairs of immune cell scores and ^18^F-FDG PET parameters (Fig. 3A, Additional file 1: Table S1). We also explored the correlation between the immune cell scores and ^18^F-FDG PET parameters in each genomic subtype group. In the TRU group, Th2, activated dendritic cell, eosinophil and mast cell scores showed weak negative correlations with the ^18^F-FDG PET parameters. In the PP group, the gamma delta T cell (Tgd) score showed a weak negative correlation with TLR. In contrast, there were pairs which showed a weak positive correlation in the PI group [Tgd vs. TLR, Tgd vs. TLG, macrophages vs. TLR and macrophages vs. TLG] (Fig. 3B-D, 3A, Additional file 1: Table S1). CYT showed weak correlations with TLR and COV (*r* = 0.308 and 0.268 and *P* = 0.01 and 0.029, respectively). In the PI group, TLR and TLG showed weak positive correlations with CYT. However, there were no significant correlations between the CYT scores and the ^18^F-FDG PET parameters in the TRU and PP groups (Fig. 4B). Overall, there was a trend toward weak negative correlations between immune cell scores and ^18^F-FDG PET parameters in the TRU and PP groups. However, a weak positive correlation was found for the PI group. This suggests that ^18^F-FDG uptake in the PI group is more affected by immune cell infiltration than in the other groups.

### Prognostic value of ^18^F-FDG PET parameters, CYT and immune cell scores

**Fig. 5:**
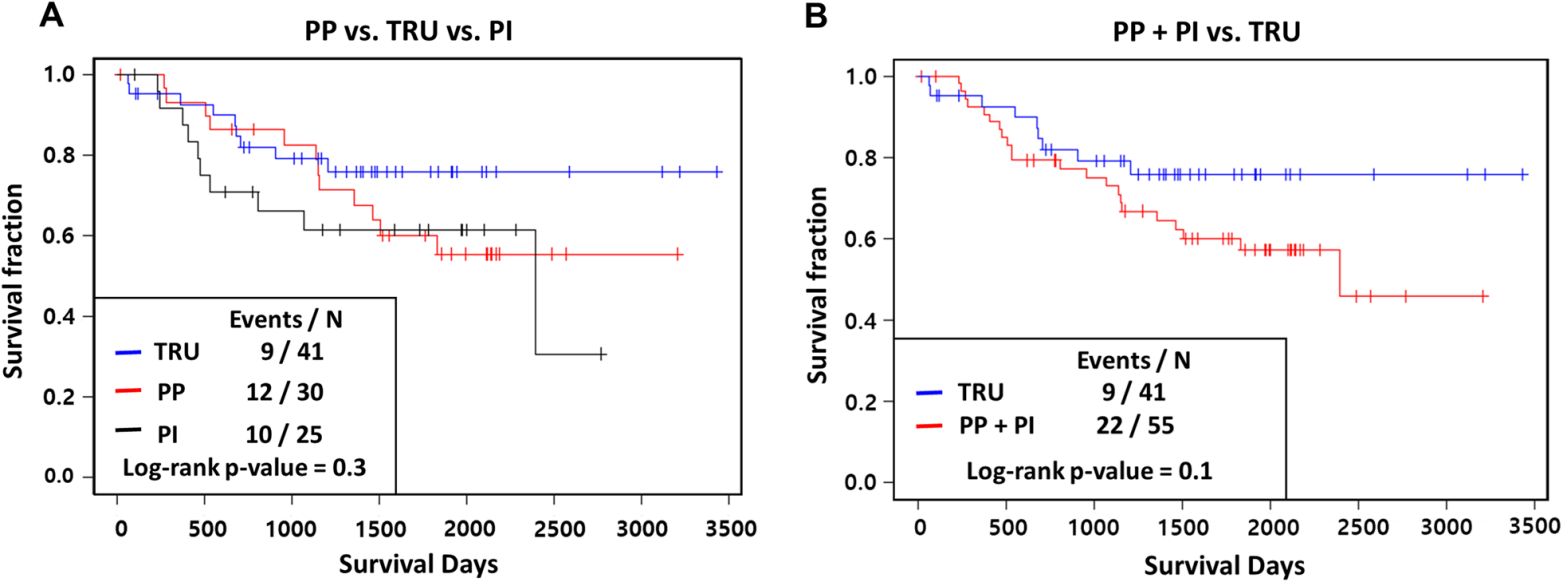
Prognostic value of molecular subtypes: **A** overall survival Kaplan–Meier curves of each group divided by the molecular subtype, and **B** PP + PI versus TRU. The boxes in each survival curve show the number of dead and the total number of patients for the groups and the p values according to log-rank tests

**Fig. 6:**
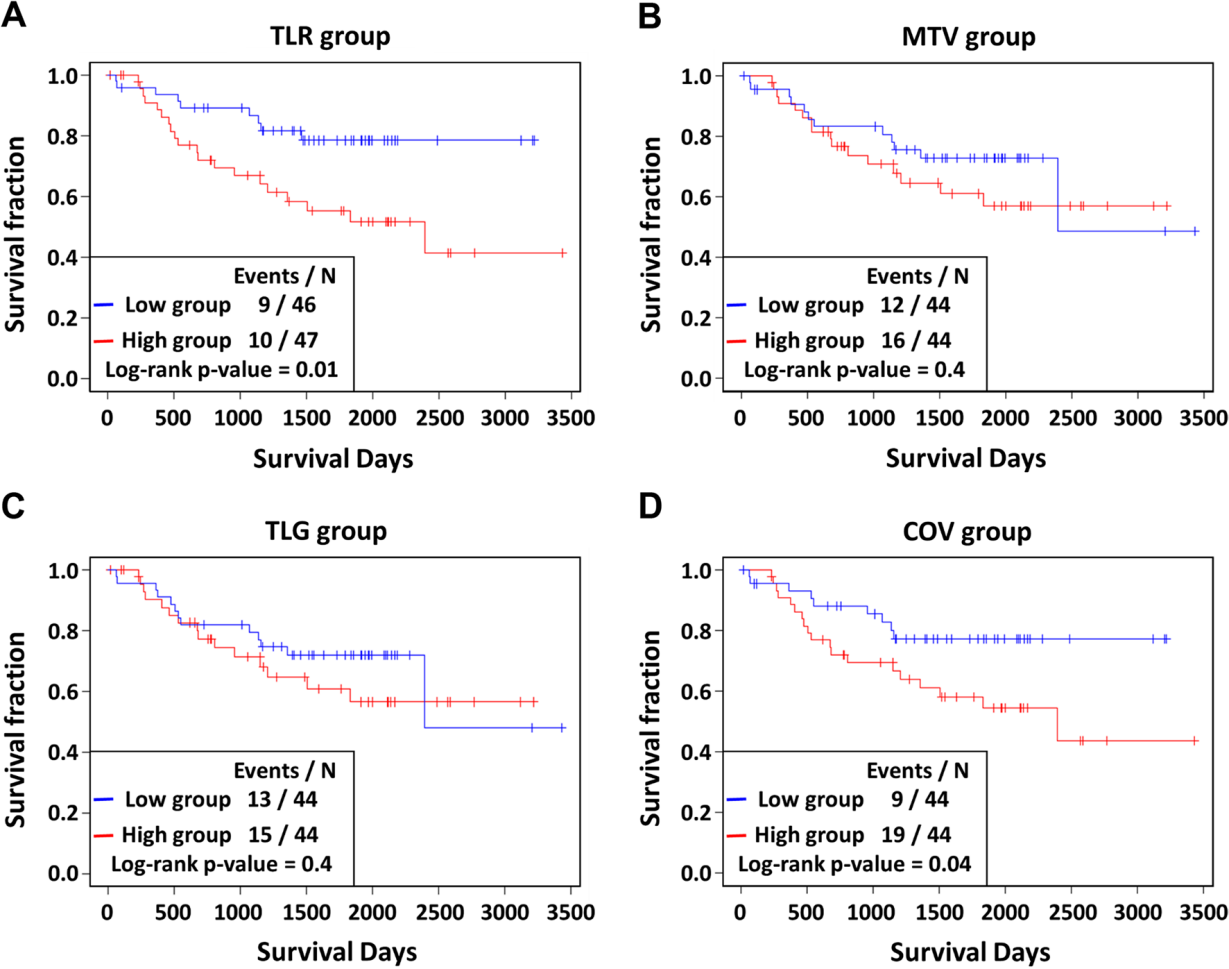
Prognostic value of ^18^F-FDG PET parameters. Kaplan–Meier curves of each **A** TLR, **B** MTV, **C** TLG and **D** COV group divided by the median values of the parameters for overall survival. Red, high group; blue, low group. The boxes in each survival curve show the number of dead and the total number of patients for groups and the p values according to log-rank tests

**Fig. 7:**
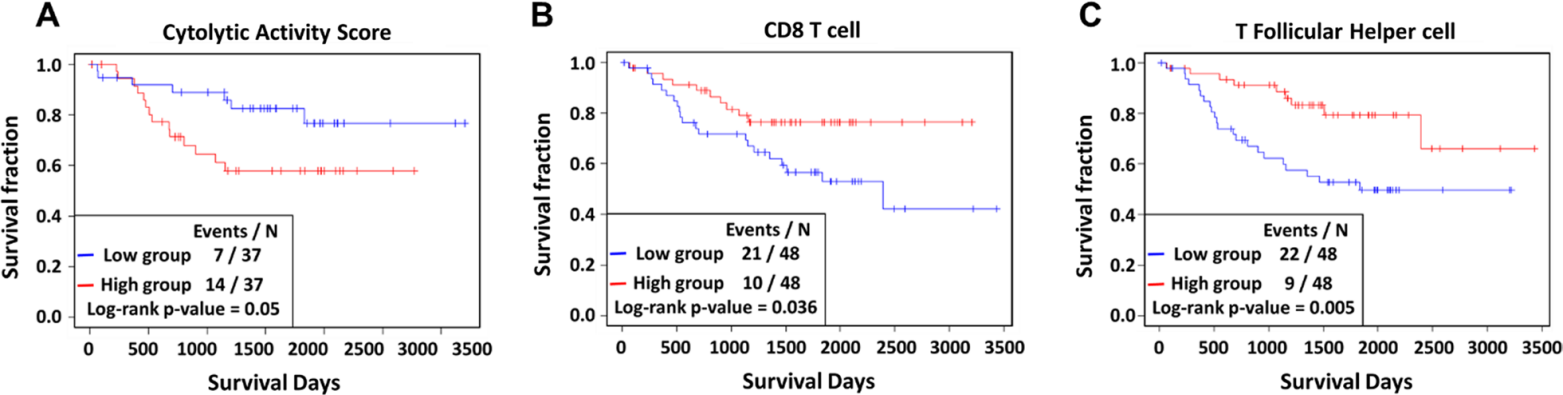
Prognostic value of immune cell scores. Kaplan–Meier curves of each **A** cytolytic activity score, **B** CD8 T cell, and **C** T follicular helper cell group divided by the median values of the parameters for overall survival. Red, high group; blue, low group. The boxes in each survival curve show a number of dead and the total number of patients for groups and the p values according to by log-rank tests

We assessed the abilities of the ^18^F-FDG PET parameters, CYT scores, immune cell scores and molecular subtypes with regard to predicting patients’ overall survival. Among the molecular subtypes, there were no significant differences in the overall survival rates between the three groups (*P* = 0.3). However, when the TRU group and the others were compared, the TRU group tended to have better clinical outcomes (*P* = 0.1) (Fig. 5), consistent with a previous report [29]. Among the ^18^F-FDG PET parameters, low TLR and low COV scores were associated with better overall survival (*P* = 0.01, and 0.04, respectively) (Fig. 6, Additional file 1: Table S2). Also, a low CYT score was associated with better prognosis (*P* = 0.05) (Fig. 7A, Additional file 1: Table S2). Among the immune scores, high T follicular helper cell (TFH) and high CD8 T cell scores were associated with better prognosis (*P* = 0.036 for CD8 T cells; *P* = 0.005 for TFH cells) (Fig. 7B-C, Additional file 1: Table S2).

**Fig. 8:**
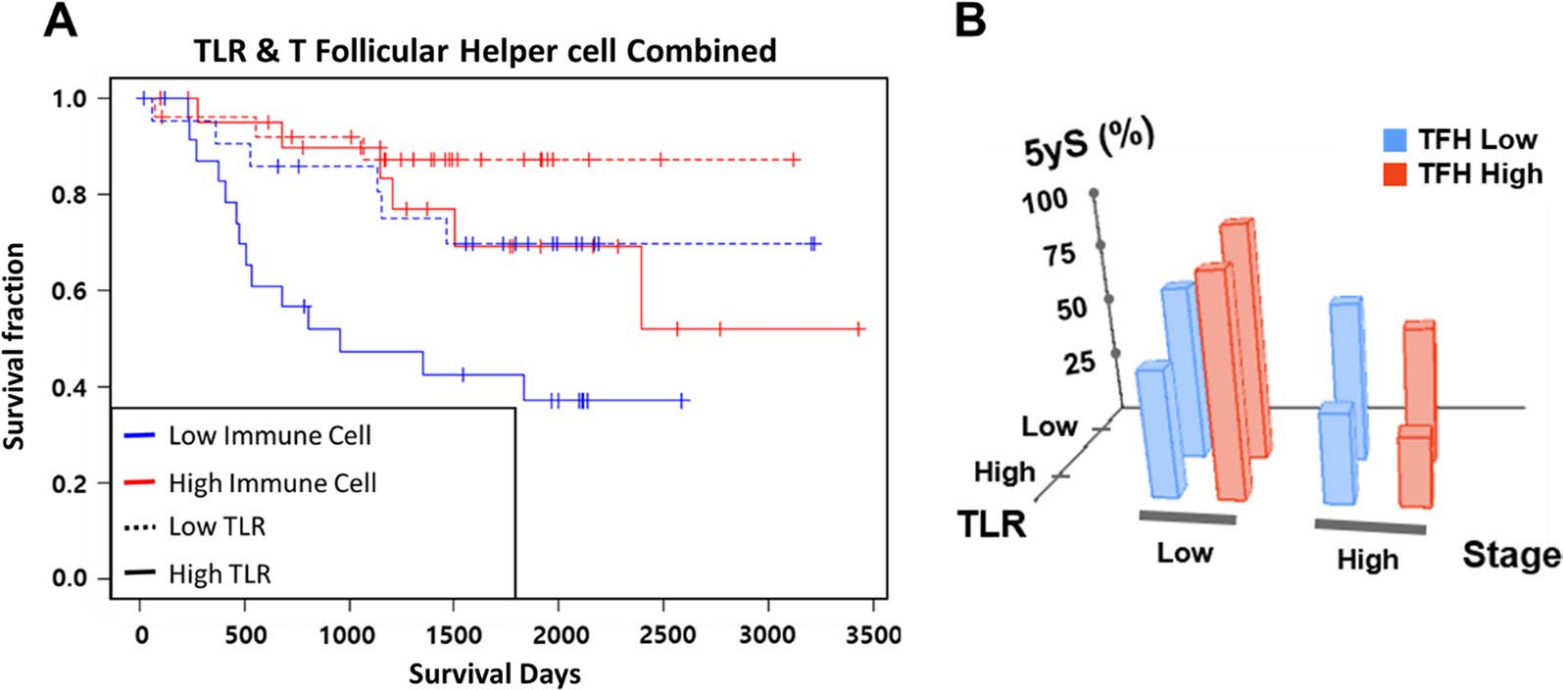
Prognostic value of combined TLR and T follicular helper cell: **A** Kaplan–Meier curves of each group divided by the median values of each of the parameters for overall survival. Red, high TFH group; blue, low TFH group; solid line, high TLR group; and dotted line, low TLR group. **B** Five-year survival rate for each group divided by the TLR, TFH and stage. TLR and TFH groups were divided by the median value. The low-stage group consists of patients who are diagnosed as having lung adenocarcinoma at stages 0 or 1 or 2, and the high-stage group consists of those at stage 3 or stage 4

Finally, we selected TLR from the ^18^F-FDG PET parameters and the TFH score from the immune cell scores, which showed the most robust parameters in their groups for predictions of clinical outcomes (lowest P value in a log-rank test) to explore the additive value of combining the immune cell score and this ^18^F-FDG PET parameter. The patients were divided into four groups, as follows: (1) patients with high TLR and high TFH scores, (2) patients with high TLR and low TFH scores, (3) patients with low TLR and high TFH scores and (4) patients with low TLR and low TFH scores. A Kaplan–Meier analysis demonstrated good stratification of the four groups, and patients in the high TLR group with a low TFH score had the worst clinical outcome (*P* = 0.002) (Fig. 8A). The five-year survival rate was visualized according to different stages in the groups according to the TLR and TFH scores (Fig. 8B). As expected, the five-year survival rate declined at higher stages and when the TLR was higher. Interestingly, the high TFH group had a longer five-year survival rate than the low TFH group at a low stage, but the trend was reversed at a high stage (Fig. 8B, Table 4). Furthermore, TLR and TFH had independent prognostic values according to a multivariate Cox regression analysis, even after adjustments with clinicopathologic features and with each other (*P* = 0.016 for TLR; *P* = 0.017 for TFH; *P* = 0.024 for adjusted TLR; *P* = 0.047 for adjusted TFH) (Table 5).

**Table 4:** Five-year survival rate according to the parameter groups

Stage	^18^F-FDG PET parameter	Immune cell score	Five-year survival (95% confidence interval)
Stage 0–2	TLR low	TFH low	0.686 (0.491–0.960)
		TFH high	0.911 (0.8–1)
	TLR high	TFH low	0.492 (0.297–0.816)
		TFH high	0.83 (0.635–1)
Stage 3–4	TLR low	TFH low	0.6 (0.293–1)
		TFH high	0.5 (0.125–1)
	TLR high	TFH low	0.333 (0.108–1)
		TFH high	0.25 (0.0458–1)

TLR low/TLR high: categorical groups divided by the median value of the tumor-to-liver SUVmax ratio, TFH low/TFH high: categorical groups divided by the median value of the follicular helper T cell score

**Table 5:** Multivariate Cox regression analysis for overall survival

Groups	Methods	Hazard ratio (95% CI)	*P* value
TLR low vs. TLR high	Unadjusted	2.645 (1.202–5.818)	0.016
	Adjusted for TFH	2.661 (1.209–5.856)	0.015
	Adjusted for age, gender and tumor stage	2.366 (1.012–1.101)	0.047
	Adjusted for TFH, age, gender and tumor stage	2.66 (1.135–6.233)	0.024
TFH low vs. TFH high	Unadjusted	0.618 (0.416–0.917)	0.017
	Adjusted for TFH	0.379 (0.172–0.835)	0.016
	Adjusted for age, gender and tumor stage	0.693 (0.450–1.067)	0.096
	Adjusted for TFH, age, gender and tumor stage	0.648 (0.422–0.995)	0.047

TLR low/TLR high: categorical groups divided by the median value of the tumor-to-liver SUVmax ratio, TFH low/TFH high: categorical groups divided by the median value of the follicular helper T cell score

## Discussion

In this study, we found that high TLR and high COV scores from among ^18^F-FDG PET parameters were associated with worse prognosis; in contrast, high CD8 T cell and TFH scores among immune cell scores were associated with favorable prognosis in patients with lung adenocarcinoma. Also, combining TLR and TFH could further stratify the prognosis of patients, and they were found to be independent prognostic features after adjustments of the clinical variables and of each individual parameter. In addition, we demonstrated that the PI group had the highest levels of anticancer immunity, ^18^F-FDG uptake and metabolic heterogeneity among the three molecular subtypes of lung adenocarcinoma.

In 2006, Hayes et al. devised lung adenocarcinoma subtypes using gene expression profiling, referring to each subtype as bronchioid (now called TRU), squamoid (now called PI) and magnoid (now called PP). They also confirmed that subtypes have prognostic importance and reported superior prognosis of the bronchioid group [30]. Wilkerson et al. found that the subtypes had different clinical profiles; TRU had the majority of females, early stage tumors and the lowest level of invasion [26], in accordance with previous [30, 31] and current studies. Moreover, they found that the TRU group had higher EGFR mutation rates, while the PP group had more KRAS and TP53 mutations than the other groups. Recently, different immune landscapes were reported among different subtypes. Faruki et al. reported that the PP subtype has low immune cell expression compared to those of the TRU and PI groups. TRU subtypes showed greater expression levels in innate immune cells, while the PI subtype showed higher expression levels of T helper 1 (Th1) and 2 cells (Th2), regulatory T cells (Treg) and cytotoxic T cells. Additionally, CTLA4 and PD-L1, the targets of immune checkpoint inhibitors, demonstrated higher levels in the PI group than in the other groups [32]. The present study also revealed that the PI group has the highest CYT scores, reflecting antitumor immunity, among the subtype groups. Furthermore, we used the benefit of ^18^F-FDG PET scans to explore the metabolic phenotypes of the subtype groups.

It has been reported that lung adenocarcinoma subtypes have different metabolism profiles [33, 34]. Our group previously demonstrated that the PI group has the highest tumor metabolism index, which is a deep-learning-based index used to predict SUVmax from transcriptomic data among the three subtypes [12]. In the present study, the prior finding is confirmed in an evaluation using an actual pair consisting of ^18^F-FDG PET and RNA sequencing data in identical patients. The PI group showed the highest TLR score among the three groups. This is in line with prior findings demonstrating that the EGFR mutation, a feature designated to the TRU subtype, is associated with a lower SUVmax value [35]. In addition, we found that the PI group had the highest COV score, which reflects the metabolic heterogeneity of the tumor. This can be explained by the fact that the PI group has the highest level of TP53 mutation and the highest mutation burden among the three groups [36].

Given that ^18^F-FDG PET uptake is primarily determined by cancer cells and infiltrating immune cells, a positive correlation between the ^18^F-FDG PET parameters and immune cell scores would be found under specific conditions; for example, 1) cancer cells are less ^18^F-FDG-avid but infiltrating immune cells are ^18^F-FDG-avid, or 2) the degrees of ^18^F-FDG avidity are similar between the infiltrating immune cells and cancer cells. Meanwhile, a negative correlation between the ^18^F-FDG PET parameters and immune cell scores would be found when 1) cancer-cell-dominant glucose metabolism leads to a restriction of certain immune cell infiltration or when 2) a certain enhanced type of immune cell infiltration suppresses tumor metabolism. We found that there were no significant correlations among all of the patients, whereas significant correlations were observed in each subtype, but with different trends. In the TRU group, the ^18^F-FDG PET parameters showed weak negative correlations with Th1, activated dendritic cell (aDC), eosinophil and mast cell scores. Additionally, in the PP group, the Tgd score showed a weak negative correlation with a TLR score. However, in the PI group, we found a weak positive correlation between Tgd and macrophage scores and the ^18^F-FDG PET parameters. Our group previously reported that the ^18^F-FDG uptake score showed an inverse correlation with the immune score in patients with low immune scores, whereas a positive correlation was found in patients with high immune scores [33]. This observation is similar to the results here, considering that the PI group reportedly has the highest immune cell scores [36] and CYT levels compared to the other groups. Relationships between metabolic parameters from ^18^F-FDG PET data and tumor-infiltrating immune cells in cancers including NSCLC have been reported [37–41]. Most studies showed that high ^18^F-FDG uptake is associated with a high level of tumor-infiltrating immune cells. Researchers have concluded that the ^18^F-FDG PET parameters may reflect the tumor microenvironment and may be a potential biomarker of immunotherapy [42–45]. However, this study represents the first dissection of ^18^F-FDG PET tumor uptake levels by means of a corresponding genomic analysis combined with tumor-infiltrating immune cell quantification in conjunction with a radiogenomic approach to explore survival outcomes.

In this study, we found that cytotoxic T cells and TFH scores were prognostic in patients with lung adenocarcinoma. Cytotoxic T cells are the key immune cells that kill cancer cells by the recognition of major histocompatibility complex class 1 molecules on the cancer cells [46, 47]. Multiple studies, including one meta-analysis, have reported that high levels of CD8 + cytotoxic T cell infiltration are associated with better prognosis in lung cancer [20, 48, 49]. TFH cells, a subset of CD4 + T cells, have an essential role in helping B cells to produce high-affinity antibodies [50]. A high level of the gene signature of TFH cells is reportedly associated with better prognosis in patients with breast cancer [51] and colorectal cancer [27]. Ma et al. also reported that the frequency of tumor-infiltrating TFH is related to better clinical outcomes in patients with NSCLC [52]. The results of the present study are in line with these previous reports, indicating that the gene signature scores of TFH and cytotoxic T cells can be considered as potential prognostic biomarkers in patients with lung adenocarcinoma.

The association between a high ^18^F-FDG tumor uptake and worse prognosis in NSCLC is consistently reported in the literature. Among ^18^F-FDG PET parameters, SUVmax, MTV and TLG have been reported to be prognostic in meta-analyses [10, 53, 54]. In the present study, we utilized TLR instead of SUVmax to minimize the effect of heterogeneous ^18^F-FDG PET data [23, 55]. In accordance with the literature, a high TLR score was associated with worse prognosis in the present study. We also found that a high COV score is related to worse prognosis. Other reports have found that high intratumoral heterogeneity is related to worse prognosis in NSCLC [19]. The present study found that known prognostic markers from ^18^F-FDG PET and immune cell scores are independently prognostic in patients with lung adenocarcinoma. This finding suggests a method for more precise prognosis predictions of lung adenocarcinoma.

Despite the intriguing nature of the findings of the present study, the findings should be carefully interpreted until they can be confirmed in independent datasets, which could not be obtained in the current study. Also, the present study was performed using a retrospective dataset which includes ^18^F-FDG PET images from multiple sites, which may also be a limitation.

## Conclusion

This radiogenomic study showed an association between ^18^F-FDG PET parameters, molecular subtypes and infiltrating immune cell scores according to ^18^F-FDG PET images and transcriptomic data in lung adenocarcinoma. We found that a high level of infiltrating immune cells was related to high ^18^F-FDG uptake in only the PI group. A high ^18^F-FDG uptake level and high heterogeneity in the ^18^F-FDG PET parameters were associated with worse prognosis, but high CD8 T cell and TFH immune cell scores were associated with better prognosis. Furthermore, TLR and TFH had additive prognostic value when combined in a survival analysis, and they could be independent predictors of prognosis with an adjustment by clinicopathologic variables and with each other.

## Supplementary Information

**Additional file 1: Table S1.** Correlation analysis of FDG parameters and immune cell scores. **Table S2.** Survival analysis.

## Data Availability

Clinical, ^18^F-FDG PET and RNA sequencing dataset can be found at the TCIA(https://www.cancerimagingarchive.net). All data analyzed during this study are included in this manuscript.

## Abbreviations

^18^F-FDG: ^18^F-fluorodeoxyglucose
NSCLC: Non-small cell lung cancer
PET: Positron emission tomography
TCIA: The Cancer Imaging Archive
TRU: Terminal respiratory unit
PP: Proximal proliferative
PI: Proximal inflammatory
CYT: Cytolytic score
SUVmax: Maximum standardized uptake value
TLR: Tumor-to-liver maximum standardized uptake value ratio
MTV: Metabolic tumor volume
TLG: Total lesion glycolysis
ROI: Region of interest
COV: Coefficient of variation
TFH: High T follicular helper cell
PRF1: Perforin-1 gene
GZMA: Granzyme A gene
