# Supplementary material for: Prognostic impact of an integrative analysis of [18F]FDG PET parameters and infiltrating immune cell scores in lung adenocarcinoma
Source: EJNMMI Res. 2022 Jun 27;12:38. doi: 10.1186/s13550-022-00908-9 (PMC9237200; doi:10.1186/s13550-022-00908-9)
Supplement: Supplementary file 1 — Additional file 1: Table S1. Correlation analysis of FDG parameters and immune cell scores. Table S2. Survival analysis. [file 13550_2022_908_MOESM1_ESM.docx]

**Supplementary Information**

This supplementary information includes whole results of correlation analysis between FDG parameters and immune cell scores. Also, it includes results of survival analysis of each FDG parameters and immune cell scores in lung adenocarcinoma patients.

**Supplementary table 1. Correlation analysis of FDG parameters and immune cell scores**

1. **Correlation coefficient of all patients**

|  | **CYT** | **B.cells** | **T.cells** | **T.helper.cells** | **Tcm** | **Tem** | **Th1.cells** | **Th2.cells** | **TFH** | **Th17.cells** | **TReg** | **CD8.T.cells** | **Tgd** | **Cytotoxic.cells** | **NK.cells** | **NK.CD56dim.cells** | **NK.CD56bright.cells** | **DC** | **iDC** | **aDC** | **Eosinophils** | **Macrophages** | **Mast.cells** | **Neutrophils** |
| --- | --- | --- | --- | --- | --- | --- | --- | --- | --- | --- | --- | --- | --- | --- | --- | --- | --- | --- | --- | --- | --- | --- | --- | --- |
| **TLR** | **0.3079** | **-0.1587** | **-0.0845** | **-0.1601** | **-0.1119** | **-0.2135** | **-0.1674** | **0.2285** | **-0.1489** | **-0.0346** | **0.0539** | **-0.0634** | **-0.1603** | **-0.0181** | **-0.1177** | **-0.0062** | **0.0501** | **-0.3123** | **-0.2634** | **-0.3509** | **-0.1510** | **-0.1925** | **-0.3683** | **-0.0929** |
| **MTV** | **0.1085** | **-0.0530** | **-0.1018** | **-0.1044** | **-0.0438** | **-0.0490** | **-0.1897** | **0.1427** | **-0.0549** | **-0.1357** | **0.0452** | **-0.1321** | **-0.1135** | **-0.1373** | **-0.0667** | **-0.0085** | **0.0286** | **-0.1762** | **-0.3333** | **-0.2493** | **-0.0528** | **-0.2158** | **-0.2277** | **-0.1125** |
| **TLG** | **0.0842** | **-0.0906** | **-0.1316** | **-0.1172** | **-0.0088** | **-0.0758** | **-0.2076** | **0.1469** | **-0.0088** | **-0.0593** | **0.0275** | **-0.1572** | **-0.1878** | **-0.1807** | **-0.0414** | **-0.0170** | **0.1021** | **-0.2230** | **-0.2964** | **-0.1418** | **-0.0033** | **-0.3128** | **-0.1096** | **-0.1205** |
| **COV** | **0.2675** | **-0.0308** | **0.0486** | **-0.0625** | **-0.1609** | **-0.1567** | **-0.0383** | **0.2244** | **-0.1973** | **-0.1721** | **-0.0568** | **0.0368** | **-0.1166** | **0.1609** | **-0.0803** | **0.0391** | **-0.0775** | **-0.1178** | **-0.1305** | **-0.3056** | **-0.2758** | **0.0345** | **-0.3782** | **0.0450** |

1. **P value of all patients**

|  | **CYT** | **B.cells** | **T.cells** | **T.helper.cells** | **Tcm** | **Tem** | **Th1.cells** | **Th2.cells** | **TFH** | **Th17.cells** | **TReg** | **CD8.T.cells** | **Tgd** | **Cytotoxic.cells** | **NK.cells** | **NK.CD56dim.cells** | **NK.CD56bright.cells** | **DC** | **iDC** | **aDC** | **Eosinophils** | **Macrophages** | **Mast.cells** | **Neutrophils** |
| --- | --- | --- | --- | --- | --- | --- | --- | --- | --- | --- | --- | --- | --- | --- | --- | --- | --- | --- | --- | --- | --- | --- | --- | --- |
| **TLR** | **0.0103** | **0.3943** | **0.9518** | **0.7080** | **0.8557** | **0.3750** | **0.3464** | **0.0122** | **0.5751** | **0.9253** | **0.5354** | **0.9143** | **0.2044** | **0.6162** | **0.8777** | **0.6125** | **0.4838** | **0.0246** | **0.1346** | **0.0108** | **0.4379** | **0.3828** | **0.0029** | **0.6020** |
| **MTV** | **0.3822** | **0.9836** | **0.3956** | **0.7024** | **0.9628** | **0.9234** | **0.1748** | **0.0551** | **0.6652** | **0.0288** | **0.7505** | **0.3567** | **0.2908** | **0.3049** | **0.7047** | **0.9725** | **0.5512** | **0.1344** | **0.0316** | **0.0761** | **0.8092** | **0.0373** | **0.1178** | **0.3561** |
| **TLG** | **0.4982** | **0.7602** | **0.4595** | **0.6368** | **0.7248** | **0.9995** | **0.1877** | **0.0767** | **0.6185** | **0.2812** | **0.8221** | **0.3330** | **0.1261** | **0.2726** | **0.7198** | **0.9917** | **0.3217** | **0.0932** | **0.0424** | **0.2704** | **0.7109** | **0.0280** | **0.3914** | **0.3629** |
| **COV** | **0.0287** | **0.9377** | **0.3178** | **0.8711** | **0.6412** | **0.6936** | **0.8199** | **0.0227** | **0.2143** | **0.3436** | **0.8044** | **0.9957** | **0.5087** | **0.1157** | **0.7184** | **0.4269** | **0.7137** | **0.4433** | **0.5828** | **0.0359** | **0.0726** | **0.5510** | **0.0008** | **0.6186** |

1. **Correlation coefficient of PP patients**

|  | **CYT** | **B.cells** | **T.cells** | **T.helper.cells** | **Tcm** | **Tem** | **Th1.cells** | **Th2.cells** | **TFH** | **Th17.cells** | **TReg** | **CD8.T.cells** | **Tgd** | **Cytotoxic.cells** | **NK.cells** | **NK.CD56dim.cells** | **NK.CD56bright.cells** | **DC** | **iDC** | **aDC** | **Eosinophils** | **Macrophages** | **Mast.cells** | **Neutrophils** |
| --- | --- | --- | --- | --- | --- | --- | --- | --- | --- | --- | --- | --- | --- | --- | --- | --- | --- | --- | --- | --- | --- | --- | --- | --- |
| **TLR** | **0.043** | **-0.071** | **-0.302** | **-0.491** | **-0.004** | **-0.220** | **-0.476** | **0.145** | **-0.027** | **-0.139** | **-0.014** | **-0.419** | **-0.604** | **-0.159** | **-0.179** | **-0.079** | **0.083** | **-0.431** | **-0.499** | **-0.478** | **-0.132** | **-0.628** | **-0.198** | **-0.161** |
| **MTV** | **0.137** | **-0.122** | **-0.189** | **-0.267** | **0.073** | **-0.047** | **-0.315** | **0.092** | **0.069** | **-0.145** | **-0.017** | **-0.335** | **-0.409** | **-0.327** | **-0.026** | **-0.044** | **0.088** | **-0.234** | **-0.453** | **-0.227** | **0.001** | **-0.566** | **0.028** | **-0.256** |
| **TLG** | **0.171** | **-0.109** | **-0.172** | **-0.225** | **0.096** | **-0.037** | **-0.340** | **0.130** | **0.081** | **-0.138** | **-0.017** | **-0.292** | **-0.378** | **-0.316** | **0.003** | **-0.018** | **0.146** | **-0.245** | **-0.448** | **-0.180** | **0.016** | **-0.544** | **0.099** | **-0.242** |
| **COV** | **-0.221** | **0.267** | **-0.029** | **-0.349** | **0.016** | **-0.076** | **-0.271** | **0.264** | **0.110** | **-0.212** | **0.149** | **-0.221** | **-0.332** | **0.200** | **-0.278** | **-0.196** | **-0.056** | **-0.301** | **-0.212** | **-0.517** | **-0.070** | **-0.297** | **-0.369** | **-0.016** |

1. **P-value of PP patients**

|  | **CYT** | **B.cells** | **T.cells** | **T.helper.cells** | **Tcm** | **Tem** | **Th1.cells** | **Th2.cells** | **TFH** | **Th17.cells** | **TReg** | **CD8.T.cells** | **Tgd** | **Cytotoxic.cells** | **NK.cells** | **NK.CD56dim.cells** | **NK.CD56bright.cells** | **DC** | **iDC** | **aDC** | **Eosinophils** | **Macrophages** | **Mast.cells** | **Neutrophils** |
| --- | --- | --- | --- | --- | --- | --- | --- | --- | --- | --- | --- | --- | --- | --- | --- | --- | --- | --- | --- | --- | --- | --- | --- | --- |
| **TLR** | **0.8698** | **0.7219** | **0.9713** | **0.4781** | **0.5119** | **0.8203** | **0.4629** | **0.1742** | **0.5668** | **0.9951** | **0.9741** | **0.6249** | **0.0077** | **0.6400** | **0.7266** | **0.7949** | **0.6970** | **0.1336** | **0.3868** | **0.1489** | **0.7075** | **0.2878** | **0.6181** | **0.6925** |
| **MTV** | **0.5989** | **0.4871** | **0.8366** | **0.9387** | **0.4031** | **0.3781** | **0.6060** | **0.2086** | **0.2107** | **0.1396** | **0.9612** | **0.6651** | **0.1388** | **0.6309** | **0.2497** | **0.7283** | **0.5911** | **0.5009** | **0.4306** | **0.5526** | **0.3416** | **0.1197** | **0.5410** | **0.6700** |
| **TLG** | **0.5111** | **0.7221** | **0.9768** | **0.8988** | **0.4085** | **0.4687** | **0.5552** | **0.2253** | **0.3587** | **0.4591** | **0.9321** | **0.6912** | **0.1116** | **0.6448** | **0.3577** | **0.7145** | **0.5041** | **0.4137** | **0.3099** | **0.6180** | **0.4862** | **0.1821** | **0.5899** | **0.6051** |
| **COV** | **0.3944** | **0.3335** | **0.5412** | **0.5836** | **0.3088** | **0.2668** | **0.7287** | **0.0833** | **0.4001** | **0.9634** | **0.2019** | **0.4180** | **0.2325** | **0.2667** | **0.6708** | **0.8094** | **0.7794** | **0.6339** | **0.9959** | **0.1591** | **0.7004** | **0.9463** | **0.1142** | **0.8454** |

1. **Correlation coefficient of TRU patients**

|  | **CYT** | **B.cells** | **T.cells** | **T.helper.cells** | **Tcm** | **Tem** | **Th1.cells** | **Th2.cells** | **TFH** | **Th17.cells** | **TReg** | **CD8.T.cells** | **Tgd** | **Cytotoxic.cells** | **NK.cells** | **NK.CD56dim.cells** | **NK.CD56bright.cells** | **DC** | **iDC** | **aDC** | **Eosinophils** | **Macrophages** | **Mast.cells** | **Neutrophils** |
| --- | --- | --- | --- | --- | --- | --- | --- | --- | --- | --- | --- | --- | --- | --- | --- | --- | --- | --- | --- | --- | --- | --- | --- | --- |
| **TLR** | **0.2455** | **-0.3952** | **-0.1568** | **-0.3329** | **-0.3363** | **-0.2715** | **-0.2062** | **-0.2442** | **-0.3035** | **-0.0435** | **-0.2129** | **-0.3542** | **-0.3617** | **0.0672** | **-0.1290** | **-0.0464** | **0.1182** | **-0.2428** | **-0.0389** | **-0.4132** | **-0.4097** | **0.1443** | **-0.2061** | **0.0487** |
| **MTV** | **0.0959** | **-0.0638** | **-0.1029** | **-0.3198** | **-0.3803** | **-0.2071** | **-0.2116** | **-0.5158** | **0.0005** | **-0.3730** | **-0.0124** | **-0.2604** | **-0.0033** | **0.1405** | **-0.3406** | **0.1687** | **0.0122** | **-0.3115** | **-0.3047** | **-0.0407** | **-0.4443** | **-0.0737** | **-0.4681** | **0.0471** |
| **TLG** | **0.2048** | **-0.2408** | **-0.1889** | **-0.3395** | **-0.4628** | **-0.2426** | **-0.2884** | **-0.4810** | **-0.0314** | **-0.2527** | **-0.1545** | **-0.3137** | **-0.1546** | **0.1820** | **-0.2870** | **0.1631** | **0.1242** | **-0.3709** | **-0.2461** | **-0.2620** | **-0.4717** | **-0.2126** | **-0.4347** | **0.0689** |
| **COV** | **0.2623** | **-0.1679** | **0.0127** | **-0.2165** | **-0.1549** | **-0.1645** | **-0.0929** | **-0.1457** | **-0.1916** | **-0.0453** | **-0.1401** | **-0.2829** | **-0.2748** | **0.1795** | **0.0220** | **0.1409** | **0.1042** | **-0.0904** | **-0.1586** | **-0.3280** | **-0.3703** | **0.0388** | **-0.1459** | **0.0645** |

1. **P-value of TRU patients**

|  | **CYT** | **B.cells** | **T.cells** | **T.helper.cells** | **Tcm** | **Tem** | **Th1.cells** | **Th2.cells** | **TFH** | **Th17.cells** | **TReg** | **CD8.T.cells** | **Tgd** | **Cytotoxic.cells** | **NK.cells** | **NK.CD56dim.cells** | **NK.CD56bright.cells** | **DC** | **iDC** | **aDC** | **Eosinophils** | **Macrophages** | **Mast.cells** | **Neutrophils** |
| --- | --- | --- | --- | --- | --- | --- | --- | --- | --- | --- | --- | --- | --- | --- | --- | --- | --- | --- | --- | --- | --- | --- | --- | --- |
| **TLR** | **0.2633** | **0.1501** | **0.6698** | **0.2793** | **0.3121** | **0.1244** | **0.2180** | **0.3359** | **0.1214** | **0.8970** | **0.0854** | **0.1861** | **0.0671** | **0.8452** | **0.6558** | **0.9068** | **0.1554** | **0.1275** | **0.5693** | **0.0189** | **0.0613** | **0.9330** | **0.1925** | **0.5104** |
| **MTV** | **0.6273** | **0.9183** | **0.8759** | **0.1301** | **0.0618** | **0.4960** | **0.3369** | **0.0041** | **0.8329** | **0.0507** | **0.7366** | **0.2118** | **0.7319** | **0.3815** | **0.0975** | **0.4354** | **0.9071** | **0.1465** | **0.0935** | **0.9245** | **0.0179** | **0.6785** | **0.0207** | **0.7745** |
| **TLG** | **0.2958** | **0.9199** | **0.7939** | **0.3292** | **0.6689** | **0.8700** | **0.4607** | **0.0053** | **0.9071** | **0.5198** | **0.0089** | **0.1076** | **0.6878** | **0.8299** | **0.3610** | **0.8195** | **0.1868** | **0.0496** | **0.0502** | **0.4471** | **0.1991** | **0.0418** | **0.0303** | **0.6378** |
| **COV** | **0.1775** | **0.6894** | **0.6402** | **0.2941** | **0.7400** | **0.4025** | **0.4463** | **0.3990** | **0.2888** | **0.9911** | **0.1606** | **0.0956** | **0.3026** | **0.4339** | **0.9524** | **0.4041** | **0.3130** | **0.2245** | **0.3446** | **0.0899** | **0.0481** | **0.8777** | **0.2662** | **0.5620** |

1. **Correlation coefficient of PI patients**

|  | **CYT** | **B.cells** | **T.cells** | **T.helper.cells** | **Tcm** | **Tem** | **Th1.cells** | **Th2.cells** | **TFH** | **Th17.cells** | **TReg** | **CD8.T.cells** | **Tgd** | **Cytotoxic.cells** | **NK.cells** | **NK.CD56dim.cells** | **NK.CD56bright.cells** | **DC** | **iDC** | **aDC** | **Eosinophils** | **Macrophages** | **Mast.cells** | **Neutrophils** |
| --- | --- | --- | --- | --- | --- | --- | --- | --- | --- | --- | --- | --- | --- | --- | --- | --- | --- | --- | --- | --- | --- | --- | --- | --- |
| **TLR** | **0.472** | **-0.204** | **0.168** | **0.162** | **0.041** | **-0.033** | **0.197** | **0.220** | **0.064** | **0.330** | **0.093** | **0.328** | **0.418** | **0.119** | **-0.003** | **-0.070** | **-0.017** | **-0.071** | **0.095** | **-0.138** | **0.213** | **0.408** | **-0.200** | **0.072** |
| **MTV** | **0.154** | **0.014** | **0.004** | **0.142** | **-0.005** | **0.153** | **-0.028** | **0.248** | **-0.090** | **-0.066** | **0.108** | **0.119** | **0.326** | **-0.069** | **-0.001** | **-0.083** | **-0.072** | **0.050** | **-0.202** | **-0.330** | **0.143** | **0.374** | **-0.304** | **-0.002** |
| **TLG** | **0.534** | **-0.036** | **0.136** | **0.186** | **0.041** | **0.135** | **0.026** | **0.240** | **0.031** | **0.053** | **0.266** | **0.219** | **0.461** | **0.102** | **0.050** | **0.089** | **0.054** | **-0.006** | **-0.005** | **-0.045** | **0.282** | **0.474** | **-0.185** | **0.097** |
| **COV** | **0.058** | **-0.239** | **-0.148** | **0.074** | **-0.167** | **-0.125** | **0.152** | **0.198** | **-0.114** | **0.085** | **-0.265** | **0.172** | **-0.036** | **-0.132** | **-0.085** | **-0.320** | **-0.203** | **-0.017** | **0.071** | **-0.102** | **0.058** | **0.391** | **-0.092** | **0.258** |

1. **P-value of PI patients**

|  | **CYT** | **B.cells** | **T.cells** | **T.helper.cells** | **Tcm** | **Tem** | **Th1.cells** | **Th2.cells** | **TFH** | **Th17.cells** | **TReg** | **CD8.T.cells** | **Tgd** | **Cytotoxic.cells** | **NK.cells** | **NK.CD56dim.cells** | **NK.CD56bright.cells** | **DC** | **iDC** | **aDC** | **Eosinophils** | **Macrophages** | **Mast.cells** | **Neutrophils** |
| --- | --- | --- | --- | --- | --- | --- | --- | --- | --- | --- | --- | --- | --- | --- | --- | --- | --- | --- | --- | --- | --- | --- | --- | --- |
| **TLR** | **0.0267** | **0.3225** | **0.4477** | **0.4052** | **0.8735** | **0.8745** | **0.3564** | **0.3320** | **0.7678** | **0.1484** | **0.6484** | **0.1573** | **0.0437** | **0.5852** | **0.9714** | **0.7155** | **0.9741** | **0.7973** | **0.6642** | **0.5999** | **0.3634** | **0.0464** | **0.3337** | **0.6654** |
| **MTV** | **0.4934** | **0.9657** | **0.8225** | **0.4870** | **0.8808** | **0.5560** | **0.8899** | **0.3086** | **0.5808** | **0.6715** | **0.6077** | **0.5441** | **0.1216** | **0.9564** | **0.9914** | **0.6463** | **0.8121** | **0.6996** | **0.3751** | **0.1718** | **0.6176** | **0.0678** | **0.1420** | **0.9129** |
| **TLG** | **0.0105** | **0.8563** | **0.4498** | **0.3703** | **0.9515** | **0.6070** | **0.9143** | **0.3216** | **0.9836** | **0.9196** | **0.2136** | **0.2906** | **0.0251** | **0.5367** | **0.8112** | **0.7582** | **0.7526** | **0.8917** | **0.9969** | **0.9226** | **0.2592** | **0.0198** | **0.3629** | **0.5938** |
| **COV** | **0.7989** | **0.2665** | **0.7013** | **0.6947** | **0.3850** | **0.5149** | **0.4982** | **0.4314** | **0.5060** | **0.8266** | **0.2379** | **0.3963** | **0.9061** | **0.7694** | **0.7127** | **0.1189** | **0.4103** | **0.9097** | **0.7263** | **0.7401** | **0.9056** | **0.0553** | **0.6190** | **0.2015** |

**Supplementary table2. Survival analysis**

|  |  |  | **Low group** |  |  | **High group** |  |  |  |
| --- | --- | --- | --- | --- | --- | --- | --- | --- | --- |
| **Category** | **Parameter** | **Median value** | **Low group**  **Mean, SD** | **No. death (%)** | **5-year survival rate (%, CI)** | **High group**  **Mean, SD** | **No. death (%)** | **5-year survival rate (%, CI)** | **Log rank**  **P value** |
| FDG | MTV | 4.83 | 1.953±1.033 | 27.27 | 0.728 (0.602-0.880) | 35.902±63.036 | 36.36 | 0.609 (0.469-0.792) | 0.4 |
|  | TLG | 11.22 | 3.950±2.623 | 29.55 | 0.719 (0.596-0.868) | 307.085±895.279 | 34.09 | 0.608 (0.464-0.798) | 0.4 |
|  | TLR | 1.543 | 0.843±0.364 | 19.57 | 0.767 (0.649-0.907) | 4.011±2.861 | 41.30 | 0.566 (0.428-0.749) | 0.01 |
|  | COV | 0.247 | 0.193±0.041 | 20.45 | 0.771 (0.649-0.915) | 0.345±0.070 | 43.18 | 0.581 (0.445-0.758) | 0.04 |
| Immune scores | CYT | 2.722 | 1.630±0.553 | 18.92 | 0.824 (0.705-0.963) | 5.932±3.918 | 37.84 | 0.576 (0.429-0.775) | 0.05 |
|  | B cell | 0.108 | -0.116±0.152 | 37.5 | 0.616 (0.487-0.779) | 0.514±0.447 | 27.08 | 0.707 (0.578-0.866) | 0.38 |
|  | T cell | -0.0316 | -0.524±0.330 | 31.25 | 0.707 (0.583-0.856) | 0.596±0.455 | 33.33 | 0.615 (0.482-0.785) | 0.39 |
|  | T helper cell | 0.038 | -0.236±0.273 | 41.67 | 0.596 (0.468-0.761) | 0.230±0.147 | 22.92 | 0.734 (0.609-0.884) | 0.093 |
|  | Tcm | 0.044 | -0.310±0.300 | 35.42 | 0.631 (0.500-0.795) | 0.230±0.139 | 29.17 | 0.690 (0.562-0.849) | 0.59 |
|  | Tem | -0.039 | -0.315±0.212 | 31.25 | 0.684 (0.559-0.837) | 0.237±0.154 | 33.33 | 0.630 (0.493-0.806) | 0.58 |
|  | Th1 cell | 0.061 | -0.123±0.165 | 37.5 | 0.611 (0.481-0.777) | 0.184±0.138 | 27.08 | 0.707 (0.578-0.865) | 0.33 |
|  | Th2 cell | 0.115 | -0.0953±0.183 | 31.25 | 0.645 (0.514-0.810) | 0.246±0.100 | 33.33 | 0.669 (0.539-0.831) | 0.91 |
|  | TFH | 0.125 | -0.145±0.202 | 45.83 | 0.527 (0.398-0.698) | 0.285±0.167 | 18.75 | 0.793 (0.671-0.937) | 0.0052 |
|  | Th17 cell | -0.0357 | -0.354±0.244 | 35.42 | 0.647 (0.520-0.805) | 0.267±0.188 | 29.17 | 0.678 (0.546-0.842) | 0.79 |
|  | TReg | 0 | -0.297±0.189 | 21.43 | 0.646 (0.540-0.774) | 0.526±0.334 | 29.41 | 0.706 (0.519-0.959) | 0.63 |
|  | CD8 T cell | 0.0473 | -0.128±0.197 | 43.75 | 0.566 (0.435-0.737) | 0.203±0.113 | 20.83 | 0.764 (0.645-0.904) | 0.036 |
|  | Tgd | 0.232 | 0.0192±0.124 | 27.08 | 0.717 (0.592-0.869) | 0.471±0.190 | 37.5 | 0.597 (0.463-0.770) | 0.25 |
|  | Cytotoxic cell | 0.0771 | -0.240±0.222 | 27.08 | 0.718 (0.593-0.869) | 0.508±0.307 | 37.5 | 0.604 (0.473-0.772) | 0.11 |
|  | NK cell | 0.0163 | -0.149±0.156 | 37.5 | 0.622 (0.494-0.783) | 0.141±0.0887 | 27.08 | 0.698 (0.566-0.862) | 0.46 |
|  | NK.CD56dim.cells | 0.0417 | -0.0495±0.0870 | 35.42 | 0.635 (0.505-0.799) | 0.165±0.0995 | 29.17 | 0.685 (0.556-0.845) | 0.65 |
|  | NK.CD56bright.cells | 0.0509 | -0.156±0.138 | 35.42 | 0.640 (0.512-0.801) | 0.218±0.156 | 29.17 | 0.674 (0.541-0.840) | 0.46 |
|  | DC | 0.131 | -0.155±0.195 | 35.42 | 0.653 (0.524-0.814) | 0.475±0.275 | 29.17 | 0.665 (0.533-0.829) | 0.73 |
|  | iDC | 0.0983 | -0.0899±0.190 | 35.42 | 0.654 (0.526-0.815) | 0.280±0.126 | 29.17 | 0.664 (0.532-0.828) | 0.73 |
|  | aDC | 0.0361 | -0.303±0.257 | 39.58 | 0.591 (0.457-0.765) | 0.376±0.209 | 25 | 0.730 (0.610-0.874) | 0.19 |
|  | Eosinophils | 0.0424 | -0.113±0.160 | 37.5 | 0.604 (0.473-0.773) | 0.165±0.0960 | 27.08 | 0.714 (0.588-0.868) | 0.29 |
|  | Macrophages | 0.0505 | -0.278± 0.323 | 33.33 | 0.653 (0.519-0.822) | 0.306±0.169 | 31.25 | 0.665 (0.540-0.820) | 0.87 |
|  | Mast.cells | 0.0745 | -0.130±0.152 | 39.58 | 0.585 (0.454-0.755) | 0.283±0.176 | 25 | 0.743 (0.622-0.888) | 0.17 |
|  | Neutrophils | 0.00711 | -0.177±0.136 | 33.33 | 0.668 (0.537-0.829) | 0.251±0.260 | 31.25 | 0.648 (0.518-0.812) | 0.84 |
